# Childhood trauma, primary emotional systems and suicidal ideation in youths with bipolar disorders

**DOI:** 10.1192/j.eurpsy.2023.1506

**Published:** 2023-07-19

**Authors:** D. Janiri, L. Moccia, S. Montanari, A. Simonetti, M. Di Nicola, G. Sani

**Affiliations:** 1 Psychiatry, Fondazione Policlinico Universitario “Agostino Gemelli” IRCCS; 2 Psychiatry, Università Cattolica del Sacro cuore, Rome, Italy

## Abstract

**Introduction:**

Bipolar disorders (BD) in youths are strongly associated with lifetime suicidal ideation. Childhood trauma is a prominent environmental stressor associated with both BD diagnosis and suicide. Primary emotional systems (PES), proposed by Jaak Pansepp, are altered in adults with BD and may contribute to suicide risk in youths.

**Objectives:**

The aim of this study was to investigate the distribution patterns of PES and childhood trauma in youths’ BD with and without suicidal ideation (BD-IS, BD-NIS).

**Methods:**

We assessed 289 participants, 103 youths with DSM-5 BD and 186 healthy controls (HCs). PES were obtained with the Affective Neuroscience Personality Scales (ANPS) and history of childhood trauma using the Childhood Trauma Questionnaire (CTQ). Suicidal ideation was assessed through the Columbia Suicide Scale for the Rating of Suicide Severity (C-SSRS). The distribution patterns of primary emotional systems and childhood trauma subtypes were tested using a multivariate analysis of variance (MANOVA), followed by a series of one-way ANOVAs and Scheffé post- hoc tests. The model was corrected for multiple comparisons. All the variables significantly different between the BD-IS and the BD-NIS group were subjected to a multivariate logistic regression model.

**Results:**

Over 48% of participants reported lifetime suicidal ideation. According to the MANOVA (Wilk’s Lambda=0.72, F=9.11, df=22, *p*<0.0001), BD-IS scored higher on the ANPS-ANGER and lower on ANPS-PLAY and ANPS-CARE than both HCs and BD-NIS, while BD-NIS reported higher and lower scores than HC. BD-IS and BD-NIS reported higher scores on ANPS-SEEK than HCs. BD-IS reported more emotional abuse, sexual abuse, physical abuse and emotional neglect than HCs, but only more emotional abuse than BD-NIS. ANPS-ANGER (OR=1.13, 95%CI=1.01-1.26, Wald=5.72) and CTQ-Emotional abuse (OR=1.26, 95% C.I.=1.04-1.52, Wald=5.72) were independent predictors of suicidal ideation in youths with BD (Nagelkerke R^2^=61%).

**Conclusions:**

Findings support the importance of assessing primary emotional systems and childhood trauma, in particular emotional abuse, in youths with BD at risk for suicide.

**Disclosure of Interest:**

None Declared

